# The Monthly Miscellany and Journal of Health

**Published:** 1846-02

**Authors:** 


### BIBLIOGRAPHICAL NOTICES.

*The Monthly Miscellany and Journal of Health*, edited by W. M. Cornell, M. D. Boston, Jan., 1846. Vol. 1, No. 1.

We have been favored with the first number of this new publication, and cannot give a better idea of its objects and character than by quoting from the introductory remarks of the editor. We welcome this new accession to the ranks of Medical Literature.—[Ed.

"We cannot say what we *may* publish, owing to the small dimensions of our work and the little experience we have had (though we have had some) in the chair editorial. But, if we had room and ability, we would make it of a miscellaneous and newspaper character, and say something of christianity, morals, education, the best methods of instruction, government both family and civil, temperance, equal rights, agriculture, manufacturing, commerce and marine interests, literature, news, reviews of books, &c. &c. We do not know that we shall speak of half these subjects, but we may say a word on all of them.

"One object to which we would devote a portion of our pages, is the means of preserving health, and thus forestalling and preventing disease. In this respect, we would, if we could, make our Journal what no one, with which we are acquainted, now is; for, while there are, at present, many able *medical* and *professional* periodicals, we know of no one specially devoted to the *preservation of health.*

"We have laid our foundation broad enough, and mean to make the work just what its title imports—*a Miscellany*, treating of things in general, as circumstances may bring them before us. In what relates to health, this Journal will always be found in opposition to empiricism in all its forms, and maintain the importance of a class of educated and thoroughly trained men for physicians. Many of the articles which we shall publish will be original; others selected."

